# Improvement of stress adaptation and insulin resistance in women with GDM by WeChat group management during novel coronavirus pneumonia

**DOI:** 10.3389/fnut.2022.1017472

**Published:** 2023-01-09

**Authors:** Yan Feng, Yuping Lv, Qi Feng, Xinna Song, Xiaoyan Li, Yongjun Wang

**Affiliations:** ^1^Department of Clinical Nutrition, Yuhuangding Hospital Affiliated to Qingdao University, Yantai, China; ^2^Department of Medical Oncology, Yan Tai Zhifu Hospital, Yantai, China; ^3^Department of General Surgery, Xi'an, China; ^4^Department of Obstetrics, Yuhuangding Hospital Affiliated to Qingdao University, Yantai, China; ^5^Department of Clinical Nutrition, The First Affiliated Hospital of Shandong First Medical University and Shandong Provincial Qianfoshan Hospital, Shandong, China

**Keywords:** novel coronavirus pneumonia (COVID-19), GDM, stress adaptation, mobile phone management, insulin resistance

## Abstract

**Aim:**

To evaluate the improvement of glycemic control and stress adaptation in patients with GDM by mobile phone WeChat management during novel coronavirus pneumonia.

**Methods:**

In this study, 75 women with GDM were included, of whom 35 were included in mobile WeChat group management as the GDM-M group and 40 as the GDM group.

**Results:**

After mobile WeChat group management for 4 weeks, E and NE were lower. MDA was lower, and SOD was higher. HOMA-IR was lower. E, NE, and cortisol were related to HOMA-IR positively, MDA was positively related to HOMA-IR, and SOD was negatively related to HOMA-IR. E and cortisol were positively related to MDA but negatively related to SOD.

**Conclusion:**

The stress adaptation disorder and insulin resistance in patients with GDM who have completed mobile WeChat group management can be improved during novel coronavirus pneumonia. Mobile WeChat management played a positive role in improving the insulin resistance of women with GDM under special circumstances, which may reduce the risk of maternal and fetal complications.

## Introduction

Novel coronavirus pneumonia is a coronavirus disease that has increased rapidly since its first identification in Wuhan, China, in December 2019 (1). The novel coronavirus pneumonia has become a public health safety event. For public safety considerations, the experts suggest that everyone should reduce their outdoors, suspend public places, and wear masks when necessary (2). The novel coronavirus pneumonia has brought panic to the masses and is a psychological stress event for the public.

The prevalence of gestational diabetes mellitus (GDM) is increasing rapidly (3). GDM-complicated pregnancies can cause adverse effects on mothers [such as the increased risk of pregnancy-induced hypertension (PIH), preeclampsia, and type 2 diabetes] and neonates [such as macrosomia, large for gestational age (LGA), and adulthood type 2 diabetes mellitus] (4). For women with GDM, daily blood glucose monitoring, regular clinical visits, and healthy lifestyle program management help control the disease and its complications (5).

During the COVID-19 pandemic, most people attempted to reduce the number of times they went out and went to the hospital for treatment to reduce their exposure to the virus (6). In contrast, quarantine or control of patients with GDM leads to reduced activities, changes in eating habits, or increased eating opportunities. So, currently, the self-management of patients with GDM is significant, especially professional and accurate self-management (7).

Several studies showed that self-management in GDM is effective (8, 9), but most of them focused on lectures or group discussions (10–12). During the COVID-19 epidemic, the self-management ability of women with GDM was particularly important, but they had limited channels to obtain professional and detailed self-management knowledge. Therefore, during the COVID-19 epidemic, there were challenges between the insufficient self-management ability of women with GDM and the good control of blood glucose.

However, our previous study reported that stress hormones were increased in women with gestational diabetes mellitus, and women with GDM have stress adaptation disorder (13). Novel coronavirus pneumonia incidents also increased the chances of psychological stress in the public. Limited by the conditions, we attempted to teach women with GDM the self-management method of blood glucose control through a special way, WeChat group management, while investigating whether only relying on mobile WeChat group management can improve the stress adaptive disorder and blood glucose of women with GDM during the COVID-19 pandemic.

## Objects and methods

### Exclusion criteria

According to the American Diabetes Association criteria with a 75-g oral glucose tolerance test (OGTT) at 24–28 weeks of pregnancy, the diagnosis of GDM was made with the cutoff value being >5.1 mmol/L at fasting, >10.0 mmol/L at 1 h, and >8.5 mmol/L at 2 h (14). The exclusion criteria were as follows: (1) pregnant women with previously known medical complications during pregnancy such as hypothyroidism, polycystic ovary syndrome, DM 1 or 2, and hyperthyreosis; (2) multiple pregnancies; and (3) women who were treated with hormones or drugs that may affect glucose and hormone concentrations.

### Patients

In this study, 75 women with GDM were included, of whom 35 were included in the WeChat group management as the GDM-Management (GDM-M) group and 40 in the GDM group. In the WeChat group, we have professional dieticians who give professional diet suggestions and answer questions from patients on time every day. Women with GDM-M received information on the risks of GDM, detailed guidance on GDM diet, and some details in activities, blood glucose and weight tests, and a 3-day diet diary that was recorded for 1 week, and this study lasted for 4 weeks. Fasting blood glucose, 2 h blood glucose, daily diet, fasting weight, and time of exercise were included in the diary. Approval was obtained from the ethics committee of Yantai Yuhuangding Hospital, and informed consent was obtained from all participants.

### Measurements

Clinical features, including the gestational age at delivery, maternal age, and pre-gestational body mass index (BMI), were included for analysis. Blood glucose was determined using Roche Automatic Biochemical Analyzer (Roche Diagnostics, Mannheim, Germany). Malondialdehyde (MDA) and anti-OS enzyme superoxide dismutase (SOD) were detected according to the instructions of the kits (Jiancheng Bioengineering Institute, Nanjing, China). Fasting insulin was tested using an electrochemical luminescence immunoassay (Roche Diagnostics, Mannheim, Germany). Plasma epinephrine (E), noradrenaline (NE), and cortisol levels were measured using radioimmunoassay (RIA) (Roche Diagnostics, Mannheim, Germany). Homeostatic model assessment of insulin resistance (HOMA-IR) was used to evaluate insulin resistance in women with GDM (15): HOMA-IR = Fins (mU/L) × FPG (mmol/L)/22.5.

### Statistical analysis

Data were evaluated using Student's *t-*test in SPSS 16.0 (SPSS, Inc., Chicago, IL, USA). Continuous variables of normal distribution data are presented as mean ± standard error. Data that were not normally distributed were log-transformed before analysis. A *P-*value of < 0.05 was considered statistically significant.

## Results

### Maternal characteristics in women with GDM

There was no significant difference in maternal age, gestational weeks, and BMI between the GDM group and the GDM-M group (Table 1).

**Table 1:** Baseline variables in two groups (*x* ± *s*).

**Groups**	***n***	**Maternal age (years)**	**Gestational weeks (weeks)**	**Pre-gestational** **BMI (Kg/m^2^)**
GDM	40	27.4 ± 2.6	26.0 ± 2.7	25.0 ± 6.8
GDM-M	35	28.0 ± 3.5	27.8 ± 3.0	24.4 ± 5.0
*t*		−0.5	−1.6	1.1
*p*		>0.05	>0.05	>0.05

### Stress hormones, oxidative stress, and insulin resistance

**Figure 1:**
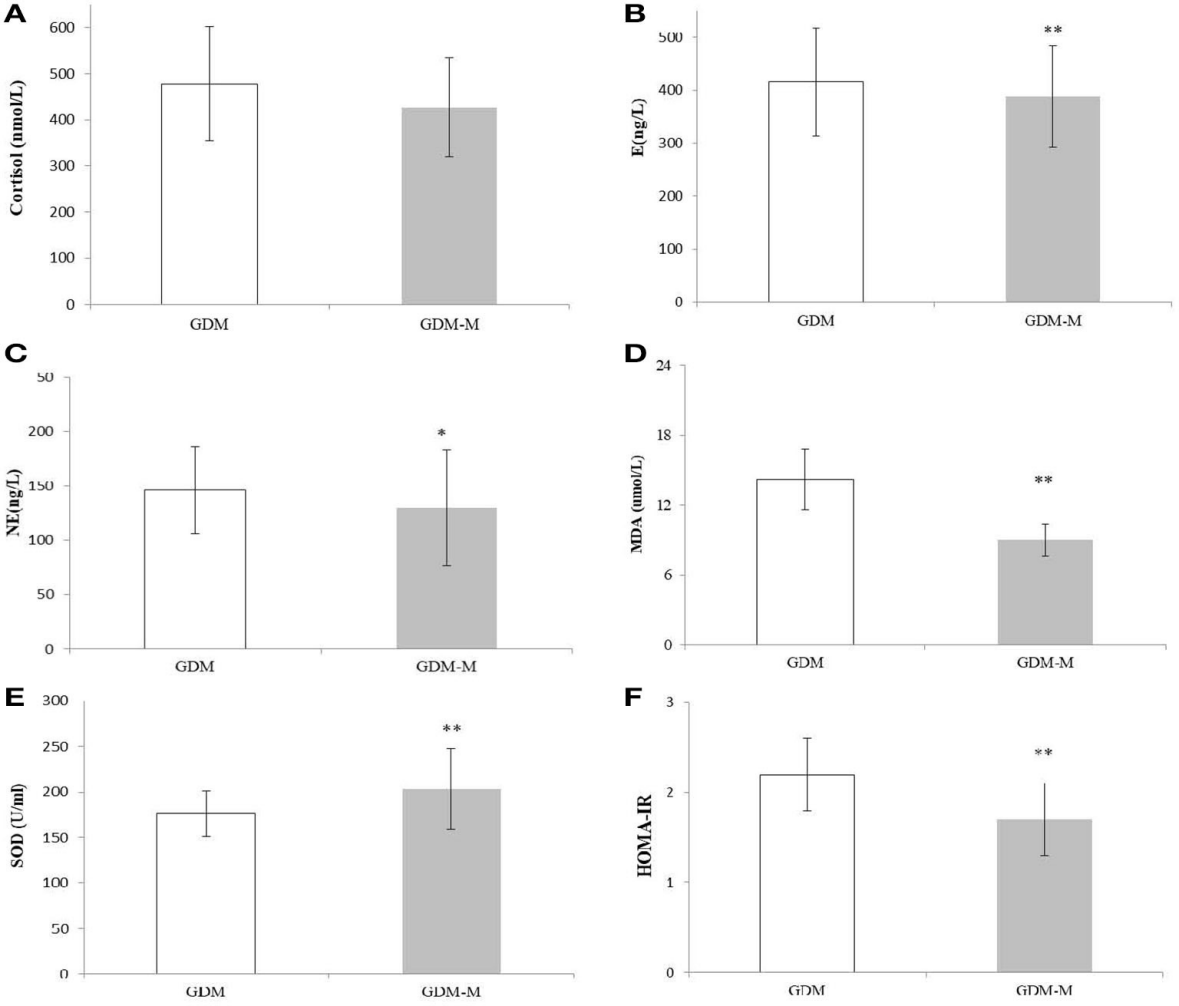
Histograms of plasma stress, oxidative stress, and glucose-related indexes—cortisol **(A)**, E **(B)**, NE **(C)**, MDA **(D)**, SOD **(E)**, and HOMA-IR **(F)** in two groups. Data are presented as mean ± SD. A two-sample *t-*test was used to determine whether the differences in the GDM-M group were statistically significant. *Indicates *P* < 0.05 vs. GDM group, and *P* < 0.01 is expressed by**. E, epinephrine; NE, noradrenaline; MDA, malondialdehyde; SOD, superoxide dismutase.

The cortisol level was slightly higher in the GDM-M group than in the GDM group, but the difference was not statistically significant (*P* > 0.05, Figure 1A). E concentration (388 ± 96 ng/L; *P* < 0.01) was lower in the GDM-M group than in the GDM group (415 ± 102 ng/L) (Figure 1B); NE levels (130 ± 53 ng/L; *P* < 0.05) were also lower in the GDM-M group than in the GDM group (146 ± 40 ng/L; *P* < 0.01) (Figure 1C). MDA was lower and SOD was higher in the GDM-M group than in the GDM group (both *P* < 0.01) (Figures 1D, E). HOMA-IR was lower in the GDM-M group (2.2 ± 0.4; *P* < 0.01) than in the GDM group (1.7 ± 0.4) (Figure 1F).

### Correlation of stress hormones and oxidative stress with HOMA-IR

In the GDM group, E, NE, and cortisol were positively related to HOMA-IR (all *P* < 0.05), MDA was positively related to HOMA-IR while SOD was negatively related (both *P* < 0.05). Only cortisol was positively related to HOMA-IR in the GDM-M group (*P* < 0.05) (Table 2).

**Table 2:** Correlation of stress hormones and oxidative stress with HOMA-IR.

**Groups**	**E**		**NE**		**Cortisol**		**MDA**		**SOD**	
	***r***	***P***	***r***	***P***	***r***	***P***	***r***	***P***	***r***	***P***
GDM	0.25	0.04	0.31	0.02	0.26	0.01	0.34	0.01	−0.22	0.03
GDM-M	0.21	0.06	0.21	0.08	0.22	0.03	0.19	0.15	−0.18	0.11

E, epinephrine; NE, noradrenaline; MDA, malondialdehyde; SOD, superoxide dismutase.

### Correlation of stress hormones with MDA and SOD

In the GDM group, E and cortisol were positively related to MDA (both *P* < 0.05), and E and cortisol were negatively related to SOD (both *P* < 0.05). In the GDM-M group, cortisol was positively related to MDA (*P* < 0.01) and E was negatively related to SOD (*P* < 0.01) (Table 3).

**Table 3:** Correlation of stress hormones with MDA and SOD.

	**MDA**						**SOD**					
**Groups**	**E**		**NE**		**Cortisol**		**E**		**NE**		**Cortisol**	
	***r***	***P***	***r***	***P***	***r***	***P***	***r***	***P***	***r***	***P***	***r***	***P***
GDM	0.35	0.02	0.18	0.22	0.33	0.00	−0.24	0.01	−0.18	0.06	−0.25	0.00
GDM-M	0.21	0.12	0.14	0.21	0.22	0.00	−0.22	0.00	−0.19	0.12	−0.14	0.22

E, epinephrine; NE, noradrenaline; MDA, malondialdehyde; SOD, superoxide dismutase.

## Discussion

To the best of our knowledge, this is the first study on stress adaptation in patients with GDM by mobile phone management during novel coronavirus pneumonia. Pregnancy for women is stressful (16). Our previous study showed that stress hormones were significantly increased in women with GDM, indicating that stress adaptation disorder occurred (13).

During the novel coronavirus pneumonia, to reduce risks, people were asked to stay at home and avoid going out. Pregnant women with GDM have been protected by their families and advised to stay at home all the time. To control blood glucose, we chose to establish a mobile WeChat management group to manage diet, blood glucose monitoring, sports education, and management for patients with GDM.

Previous studies showed that, based on the mobile WeChat group to manage blood glucose, most of them can achieve relatively ideal results in terms of blood glucose indicators (17, 18). Our tertiary hospital has professional clinical nutrition experts, obstetricians, and nursing teams and has advanced GDM management and education experience. Patients with GDM have high compliance, and it is easy to achieve ideal management results. However, during the epidemic, the only method to manage the blood glucose of patients with GDM is by establishing a WeChat group using mobile phones.

Stress is a matter of debate (19, 20). It is defined as the nonspecific response of the body to any stressor (21–23). However, prolonged stress-related psychophysiological alterations may increase the risk of functional or mental disorders, induce cytological effects, and lead to diseases (16, 24).

Stress could activate the hypothalamic-pituitary-adrenal (HPA) axis, induce cytological effects, and lead to diseases (25). Cortisol is the most frequently used biomarker of the physiological stress responses of the HPA axis. Hosler's study showed that stress is a potentially chronic, long-term, and rarely detected risk factor for metabolic disturbances, and more studies on stress exposure and psychological stress as GDM risk factors are needed (26).

Our results showed that cortisol was slightly lower in the GDM group, but the difference was not statistically significant. E and NE concentrations decreased, indicating that stress adaptation disorder decreased after mobile phone intervention, which means that, using WeChat group management, some of the stress hormones of patients with GDM decreased and the stress adaptation disorders of patients with GDM improved. E, NE, and cortisol were positively related to HOMA-IR, which indicates that, if the stress adaptation disorder of patients with GDM is improved, their insulin resistance will also decrease.

Throughout the experiment, all GDM-M participants considered choosing the right diet, adequate physical exercise, accurate blood glucose monitoring, and a standard diet diary. During the epidemic period, the level of psychological stress in the public increased (27, 28). We believe that the stress adaptation disorder of patients with GDM will further increase. This hypothesized pathway leads to the development of GDM, including psychological stress responses and stress adaptation disorder.

Pregnancy is considered a state of enhanced oxidative stress (29–31). A higher level of oxidative stress could lead to pathological pregnancies, including gestational diabetes mellitus (GDM) (32). It has been confirmed that oxidative stress plays an important role in the etiology of congenital malformations in animal models of diabetes (33).

Stress hormones including E and cortisol were positively correlated with MDA and negatively correlated with SOD, suggesting that the mechanism of stress adaptive disorder was positively related to oxidative stress injury, which was consistent with our previous research findings (13).

Our results showed that MDA was decreased and SOD was increased in the GDM-M group, and MDA and SOD were positively and negatively correlated with HOMA-IR, respectively. These findings indicate that oxidative stress impairment was lower after mobile phone management, suggesting that the decrease in oxidative stress injury in patients with GDM could improve the stress adaptive disorder, thus improving the insulin resistance of patients with GDM.

Our study has two limitations. First, there was a significant difference at baseline between the GDM-M and GDM groups. Patients with GDM who are willing to join the mobile WeChat management group may have a significant influence on compliance, providing more positive findings. Second, our study reports only the short-term effects and cannot conclude long-term mobile phone management.

Our previous interventional experimental study found that whey protein preloading can alleviate the stress adaptation disorder of patients with GDM and improve blood glucose (34). We pondered if WeChat group management plus whey protein preload will have a more surprising effect on these patients. In the future, we will continue to carry out a series of studies.

## Conclusion

In summary, during novel coronavirus pneumonia, patients with GDM who have complete mobile phone management can improve their stress adaptation disorder and play a positive role in improving insulin resistance in women with GDM under special circumstances, especially during the epidemic, which may reduce the risk of maternal and fetal complications. Compared with some diabetes-related applications, a study on the long-term effects and cost-effectiveness of blood glucose management in patients with GDM based on mobile phone WeChat management group is warranted, especially during the novel coronavirus pneumonia or other infectious diseases.

## Data availability statement

The raw data supporting the conclusions of this article will be made available by the authors, without undue reservation.

## Ethics statement

The studies involving human participants were reviewed and approved by Yantai Yuhuangding Hospital Committee. Informed consent was obtained from all participants.

## Author contributions

YF and YW designed the study and wrote the manuscript. YL and QF performed the data analysis. XL and XS provided study oversight. All authors approved the final manuscript.
